# Respiratory infection of mice with mammalian reoviruses causes systemic infection with age and strain dependent pneumonia and encephalitis

**DOI:** 10.1186/1743-422X-10-67

**Published:** 2013-03-01

**Authors:** Lianne Gauvin, Steffany Bennett, Hong Liu, Mansoureh Hakimi, Michael Schlossmacher, Jay Majithia, Earl G Brown

**Affiliations:** 1 Department of Biochemistry, Microbiology and Immunology, Faculty of Medicine, University of Ottawa, 451 Smyth Rd, Ottawa, Ontario K1H 8M5, Canada; 2 Emerging Pathogens Research Centre, University of Ottawa, 451 Smyth Rd, Ottawa, Ontario K1H 8M5, Canada; 3 Department of Cellular and Molecular Medicine, Faculty of Medicine, University of Ottawa, 451 Smyth Rd, Ottawa, Ontario K1H 8M5, Canada; 4 Program in Neuroscience, Ottawa Hospital Research Institute University of Ottawa, 451 Smyth Road, RGN #1412, Ottawa, ON K1H 8M5, Canada; 5 Division of Neurology, The Ottawa Hospital, University of Ottawa, 451 Smyth Road, RGN #1412, Ottawa, ON K1H 8M5, Canada

**Keywords:** Mammalian reovirus, Pathogenesis, Pneumonia, Systemic infection, Intranasal infection, Serotype 3 Dearing, Serotype 1 Lang, Encephalitis, Immunofluorescence, Liver, Lung, Suckling mouse, Viremia

## Abstract

**Background:**

Because mammalian reoviruses are isolated from the respiratory tract we modeled the natural history of respiratory infection of adult and suckling mice with T1 Lang (T1L) and T3 Dearing (T3D) reoviruses.

**Methods:**

Adult and suckling Balb/c mice were infected by the intranasal route and were assessed for dose response of disease as well as viral replication in the lung and other organs. Viral antigen was assessed by immunofluorescence and HRP staining of tissue sections and histopathology was assessed on formalin fixed, H + E stained tissue sections.

**Results:**

Intranasal infection of adult mice resulted in fatal respiratory distress for high doses (10^7^ pfu) of T1L but not T3D. In contrast both T1L and T3D killed suckling mice at moderate viral dosages (10^5^ pfu) but differed in clinical symptoms where T1L induced respiratory failure and T3D caused encephalitis. Infections caused transient viremia that resulted in spread to peripheral tissues where disease correlated with virus replication, and pathology. Immunofluorescent staining of viral antigens in the lung showed reovirus infection was primarily associated with alveoli with lesser involvement of bronchiolar epithelium. Immunofluorescent and HRP staining of viral antigens in brain showed infection of neurons by T3D and glial cells by T1L.

**Conclusions:**

These mouse models of reovirus respiratory infection demonstrated age and strain dependent disease that are expected to be relevant to understanding and modulating natural and therapeutic reovirus infections in humans.

## Introduction

Although mammalian orthoreovirus infection is not associated with disease, viruses are isolated from the respiratory and enteric tracts of humans and animals [1-4]. Natural infection with reoviruses may therefore involve either the nasal and/or oral routes of transmission. Recently serotype 3 strain Dearing (T3D) has been shown to possess oncolytic properties and is currently in clinical trials in humans raising fundamental questions regarding the natural history and normal patterns of reovirus infection in humans [5-7] as well as animal models.

Depending on the reovirus strain the suckling mouse can be infected by injection to cause encephalitis [8,9] and myocarditis [10-12], and the oral route of infection has been shown to differ in effectiveness among reoviruses. In particular type1 strain Lang (T1L) and murine type 3 isolate Clone 9, can establish infection in suckling mice but not adult mice via the oral route [13-15], and high dosages of type 3 strain Dearing (T3D) are required for infection of mice via the oral route in both suckling and adult animals [16,17].

Although the prototype T1L and T3D viruses were initially isolated from stool samples of human infants, clinical specimens of reoviruses are routinely isolated from human respiratory tracts [1]. However, experimental data regarding mammalian reovirus respiratory infections are limited. Intranasal infection of adult volunteers has been demonstrated for all 3 serotypes of reoviruses (T1L, T2 Jones, and T3D) indicating that humans are infectable by the respiratory route using high dosages (10^7^ pfu) of virus with the induction of relatively mild clinical signs of respiratory infection for some patients [3]. The respiratory route, has been less well studied in the mouse, however models of T1L induced respiratory disease are described in specific strains of mice where CBA/J mice induced acute respiratory distress (ARD) which has been studied primarily from the standpoint of the immune responses to infection [18-20] but has also been shown to cause systemic spread via the blood to the spleen and intestine [21]. Pneumonia is also induced by T1L in other strains of mice (CD-1, Balb-c, and C3H) but with less severe pathology [22]. Although respiratory infection of mice with strains other than the prototype T1L reovirus has not been described, reovirus respiratory infection has been studied for T1L and T3D in adult rats which resulted in fatal pneumonia for T1L infections [23,24]. Recently lung infection of CBA/J mice via the intranasal route with T1L has been shown to be enhanced by prior uncoating with protease treatment [25].

Given that nasal infection of suckling mice has not been described for reoviruses we assessed the response of adult and suckling Balb-c mice to intranasal infection with T1L and T3D reoviruses. We found that both reoviruses establish infections in adult and suckling mice with high level replication in the lung as well as spread to involve peripheral organs. Fatal disease was associated with high dosage of T1L but not T3D in adult mice and with moderate dosages of T1L and T3D in suckling mice. The pattern of symptoms, viral replication and histopathology were consistent with fatal T1L pneumonia and T3D encephalitis. These data define the basic virological and pathological response to respiratory infection with reoviruses, indicating that both T1L and T3D can readily establish infection by the respiratory route but that these viral types differ in their tropism. T1L and T3D were pneumotropic but differed in extent. T3D also demonstrated fatal neurotropic infection following respiratory infection of new-born but not adult animals. Viral spread appeared to occur via the blood stream for both T1L and T3D viruses.

## Methods

### Viruses and cells

T1L and T3D prototype strains and L929 cells were originally obtained from Dr. B.N. Fields laboratory. Viruses were propagated and titrated in mouse L 929 cells grown in MEM plus 5% fetal bovine serum and penicillin and streptomycin as described previously [26].

### Mouse infections and titrations

Adult female 4–6 week old Balb-c mice (Charles River, St. Hyacinthe, Quebec) were anaesthetized with halothane (3% in oxygen) before infection by application of virus suspended in 0.05 ml PBS onto the nose pad. This method introduces virus into the lung as evident by distribution of trypan blue dye into lung tissues of anaesthetized mice that were euthanized without recovery from anaesthetization (data not shown). Suckling Balb-c mice were similarly infected at 2 days of age with 0.01 ml volumes of virus. Survival was monitored for 30 days. Viral growth and pathology were determined on tissues collected from mice euthanized by CO_2_ narcosis. Blood was collected by cardiac puncture. Organs from duplicate animals were collected at each time point, suspended in 9 volumes of PBS and disrupted by sonication for 4 minutes on ice using a microprobe and the Model F60 Fisher Scientific sonicator at power setting 5–7 before duplicate titrations by plaque assay in L929 cells to yield 2 technical replicate titrations for each experimental replicate. Infectious titres were calculated per gram of tissue. Data was plotted as mean values with variation shown as ± 1 standard error.

### Animal ethics approval

This study was carried out in accordance and compliance with the guidelines of the Canadian Council on Animal Care (CCAC) as outlined in the Care and Use of Experimental Animals, Vol.1, 2nd Edn. (1993), which are recognized as “best-practices” by the International Council for Laboratory Animal Science (ICLAS). The protocol was approved by the University of Ottawa Animal Care Committee (Protocol Number: BMI-85).

### Histopathology

Adult mice were perfused with PBS and then formalin (3.75% formaldehyde in PBS) before dissection and removal of organs for immersion in formalin for 24 hr, paraffin embedding, sectioning and hematoxylin and eosin staining. Suckling mice were not perfused before fixation but instead were dissected and placed in formalin for 24 hr fixation with the exception of lungs that were inflated with formalin under 25 cm water pressure for 15 minutes before further fixation and processing as described for adult tissues. Horse radish peroxide (HRP) staining of formalin fixed paraffin embedded T1L and T3D infected brain tissues was done as described previously [27]. Images were collected using the 10X objective using an Olympus BX50 microscope.

### Frozen sectioning and immunofluorescent staining

Animals were sacrificed and perfused as described above before excising tissues that were snap frozen on dry ice in OCT compound (Fisher Scientific, Ottawa) for sectioning as previously described [28]. Tissue sections were fixed with cold acetone before immunofluorescent staining using the corresponding rabbit immune serum produced against purified T1L or T3D viruses. Immune sera were preadsorbed with acetone extracted, powdered mouse tissues (10% (w/v) tissue mixed with serum for 16 hr at 4 C). Primary antibodies were diluted (1/800) in 0.3% BSA in PBS and incubated with sections for 0.5 hr followed by 3 × 5 min washes in PBS. The secondary antibody was CY3 conjugated donkey anti rabbit IgG (1/800 dilution) (Jackson Labs, Maine) that was reacted as for the primary antibodies. After the last PBS wash the sections were incubated with DAPI (1 ug/ml) for 2 minutes to stain nuclei. Images were collected using an epifluorescent Leica DMXRA 2 microscope (Leica Mississauga ON) using a Hamamatsu ORCA ER camera (Hamamatsu Corp., Bridgewater, NJ) running OpenLab v3.17 software (Improvision, Lexington, MA). Images were subsequently processed using Adobe Photoshop (Adobe Systems, San Jose CA).

### Statistical analysis

Statistical analysis employed the single sample or paired t test as indicated, using the Microsoft Office 2007 XL program.

## Results

As the suckling mouse model of mammalian reovirus infection has been extensively studied following inoculation via the oral and injection routes, we set out to extend this analysis to the comparison of infection in adults and suckling mice following respiratory inoculation. Intranasal instillation of virus inoculum into anaesthetized mice results in the introduction of virus into the upper respiratory tract and lung.

### Age and virus dependent pneumonia and encephalitis in response to intranasal infection

**Figure 1:**
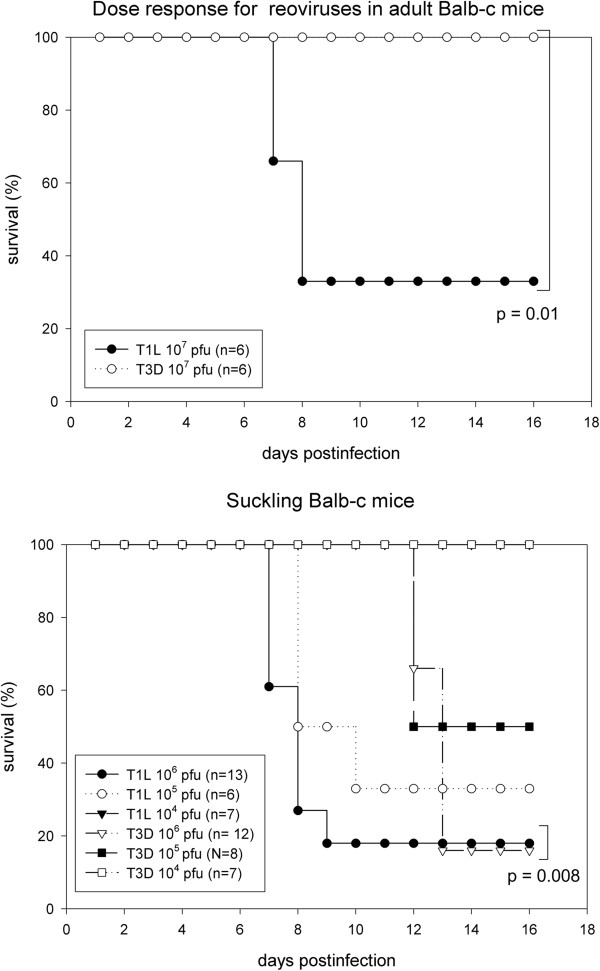
**Age and virus dependent fatal dose response of reovirus T1L and T3D infection in adult and suckling Balb-c mice.** The number of animals monitored in each group is shown as values of n. Top panel. Adult mice were intranasaly infected with 10^7^ pfu of either T1L or T3D and monitored for 30 days. Lower dosages did not induce fatality (not shown). Bottom panel. Suckling mice at 2 days of age were intranasaly infected with graded dosages of either T1L or T3D and monitored for 30 days. The times to death were significantly different using the students t test for the comparisons indicated in brackets.

Adult Balb-c mice (4–6 wk old) were infected with graded dosages of T1L or T3D virus and observed for 30 days. The highest dose of T1L (1 × 10^7^ pfu) induced signs of disease and resulted in the death of 4 of 6 mice commencing 7 days post infection (Figure 1) that was associated with piloerection, hunching and respiratory distress that was consistent with pneumonia. The same dose of T3D did not induce any detectable signs of disease and resulted in a statistically significant increased survival (time of death greater than 16 days, p = 0.01 by student’s t test) indicating a distinct difference in disease syndrome following respiratory infection with these viruses (Figure 1).

In contrast to adult mice, both T1L and T3D viruses resulted in lethal infections in suckling mice with similar dose responses (LD_50_ of 10^5^ pfu), however the time to death and the clinical symptoms of infection were significantly different (t test, p = 0.0082) (Figure 1). Suckling mice infected with T1L developed respiratory crackling sounds on day 1 or 2 that progressed to severe respiratory distress and death by day 7. Suckling mice infected with T3D also manifested respiratory crackling on day 2 but this resolved by day 8 and was followed by signs of neurological dysfunction and death commencing on day 11. Neurological symptoms included tremors with darting and/or agitated behavior, spinning or turning, ataxia, and asymmetric gait. These experiments showed that respiratory infection with T1L and T3D not only induced different types of diseases in suckling mice but also differed in their ability to induce disease in adult mice.

### Viral replication in the lung and spread to peripheral organs

**Figure 2:**
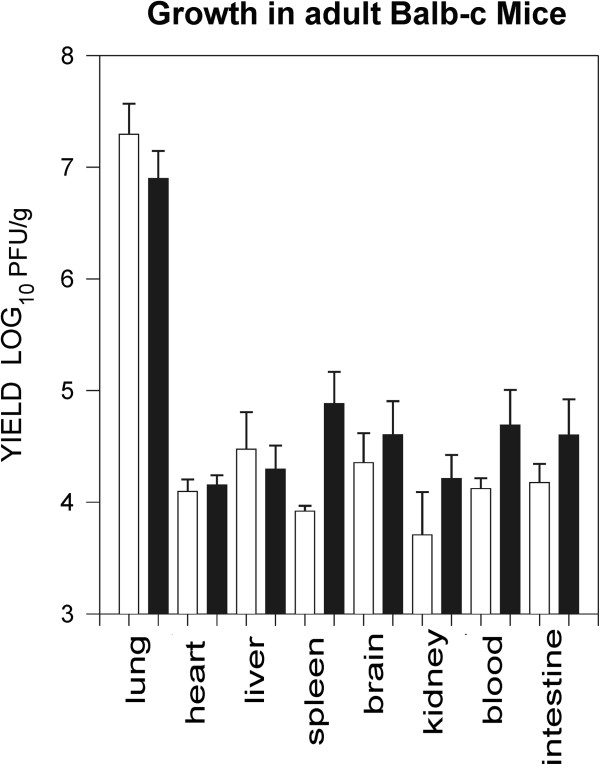
**Reovirus T1L and T3D replicates in lung and peripheral tissues following respiratory inoculation.** Groups of 2 adult mice were infected intranasaly with 10^5^ pfu of either T1L (white bars) or T3D (black bars) before collecting various organs 2 days pi. The yield of infectious virus was determined for each mouse and is shown + 1 SE for each virus and tissue type. The bars for T1L infected animals are open and for T3D are closed.

Infection of adult mice with 10^7^ pfu of T1L was associated with fatal disease in contrast to T3D that did not produce clinical disease, so it was not clear whether T3D was establishing an infection in adult mice. To further characterize reovirus respiratory infection and determine whether differences in ability to infect or replicate in tissues was responsible for the differences in disease response between these viruses, the level of infectious virus was determined for different tissues 2 days after infection with 10^5^ pfu of each virus. Both T1L and T3D yielded high infectious titres in the lung (approximately 10^7^ pfu/g) and virus was observed to have spread to other tissues to yield 100–1000 fold lower levels of virus in the heart, liver, spleen, brain, kidney, blood and intestine (Figure 2). We cannot exclude the possibly that the virus detected in the intestinal samples was from infection via the gastric route as a consequence of respiratory inoculation versus dissemination from the lung and thus the significance of intestinal virus awaits further characterization. The presence of 10^4^ pfu/g virus in blood on day 2 following infection with either T1L or T3D indicated that reovirus was employing the hematogenous route for spread in the adult mouse.

**Figure 3:**
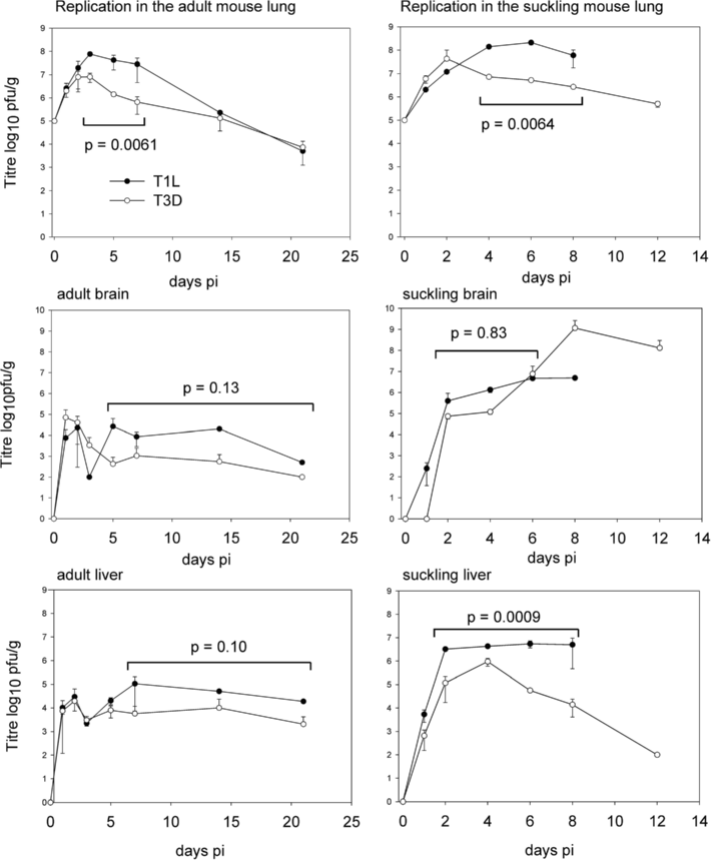
**Yield of infectious virus in lung, brain and liver of adult and suckling mice.** Groups of 2 adult or 2 day old suckling mice were infected intranasaly with 10^5^ pfu of either T1L or T3D before assaying infectious virus in lung, brain and liver. The titres represent the mean of 2 animals which where titrated in duplicate for all time points except for the later 15 and 21 day time points for the T1L infections that represent single animals with duplicate titrations. The yield of infectious virus is shown + 1 SE for each time point. The yield values are closed for T1L and open for T3D. The p statistics comparing yields of virus for T1L versus T3D are indicated for groups of values in brackets (n = 6–8) that were determined by the paired students t test with significant differences considered as p ≤ 0.05 for adult and suckling lung as well as sucking liver.

**Figure 4:**
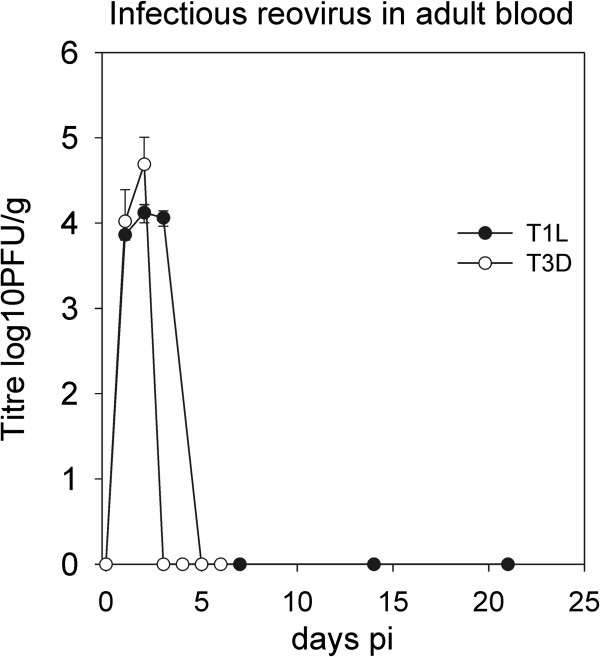
**Respiratory infection of adult mice results in transient viremia.** Groups of 2 adult mice were infected intranasaly with 10^5^ pfu of either T1L or T3D before assaying infectious virus in the blood at various times pi. The yield of infectious virus is shown + 1 SE for each time point. The yield values are closed for T1L and open for T3D.

To more fully assess the infection process, the time course of viral replication was measured in the lung as well as the brain and liver of adult mice. Growth of both T1L and T3D were similar for the first 2 days in lung tissues, at which time T3D had attained a maximum titre (Figure 3). T1L continued to replicate to reach a maximal titre on day 3 of 7.6 × 10^7^ pfu, which was 10 fold higher than T3D. The higher yield of T1L than T3D infected mice was statistically significant when compared over days 3, 5, and 7 by paired t test for n = 6 samples (p = 0.0061). Replication in the liver and brain was to lower levels than the lung but was statistically similar for both viruses (p ≥ 0.05 by paired t test n = 6–8). Virus was found in the blood for the first 2 and 3 days of infection for T3D and T1L respectively, after which infectious virus was undetectable (< 10^2^ pfu/g) (Figure 4).

The yield of infectious virus was also measured for suckling mice infected via the respiratory route with the same dose of virus as employed for adult mice (10^5^ pfu). In the lung and liver, reovirus T1L grew to significantly higher levels than T3D, p = 0.0064 and 0.0009, for days 4–8 and 2–8 respectively (by paired t test, n = 6–8) (Figure 3). In the brain T1L and T3D produced similar levels of virus until day 6 where after T3D grew to produce 1000 fold more virus on day 8 pi. Virus yield comparisons were not determined beyond day 8 because T1L produced lethal responses by day 7 pi. The high T3D titre in the brain was consistent with neurological signs of encephalitis in T3D infected suckling mice. The higher yield of T1L relative to T3D in suckling mouse lungs was also consistent with increased respiratory damage observed as respiratory distress. Both T1L and T3D grew to higher titres in the suckling mouse relative to the adult animal (Figure 3) which was consistent with their greater virulence in suckling animals.

### Immunofluorescent staining of mouse lungs demonstrated infection of alveoli

**Figure 5:**
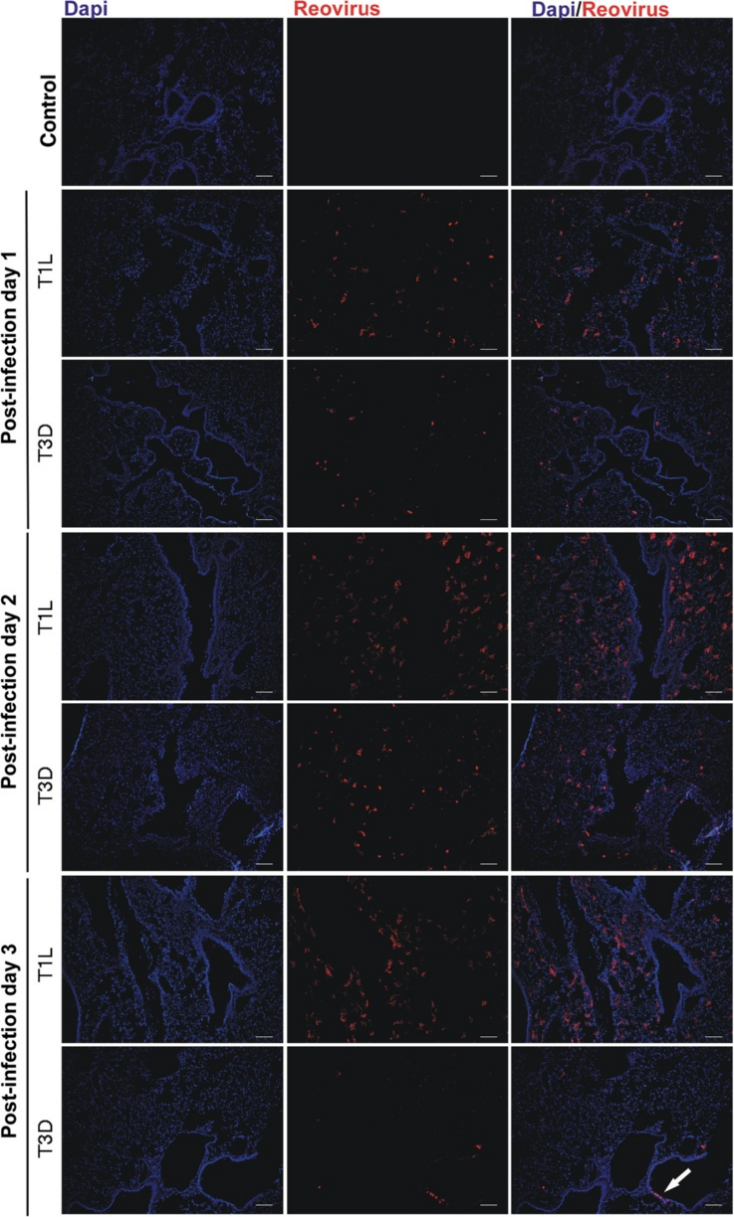
**Time course of immunofluorescent staining of virus infected adult lungs.** Groups of 2 adult mice were infected intranasaly with 10^7^ pfu of either T1L or T3D before fluorescent antibody staining of frozen lung sections at 1, 2, or 3, days pi. T1L and T3D antigens were stained with virus specific antiserum as the primary antibody followed by CY3 labeled donkey anti-rabbit IgG and are colored in red. Cell nuclei were stained with DAPI and are shown in blue, either alone or merged with the CY3 stained images. Sagital sections show extensive staining in alveolar regions as well as sparse staining of epithelium in the bronchiolar airways (arrow) restricted primarily to T3D infection. Uninfected control lungs are shown as negative staining controls. Size bars are 100 μM.

**Figure 6:**
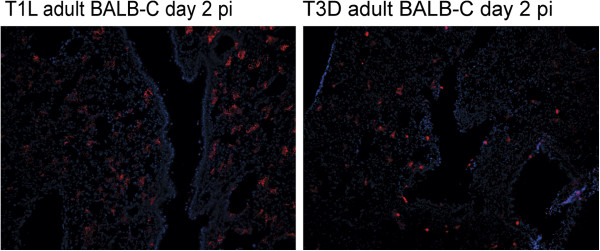
**High magnification images of T1L and T3D infected adult lungs.** The 2 day post infection images shown in Figure 5 are enlarged here to demonstrate more clearly the presence of reovirus antigens in alveolar tissues. Size bars are 100 μM.

Adult mice were infected with 10^7^ pfu of both strains of reovirus and monitored for viral antigen production in the lungs by immunofluorescent staining using type-specific antivirus rabbit serum (Figure 5). One day after infection viral antigen was prominent in lungs as multiple small patches of infection in the alveolar regions for both T1L and T3D infections. The number and size of infected sites had increased by day 2 (shown enlarged in Figure 6) but stabilized or decreased by day 3. The extent of viral staining reflected the viral growth in lungs where peak viral load and antigen staining were seen at days 2 and 3 for T3D and T1L respectively. There were very few patches of viral antigen present in bronchial epithelium which was primarily seen as individual cells or small patches of infected cells suggesting that bronchial epithelium was not a major site of infection for either T1L or T3D reoviruses.

**Figure 7:**
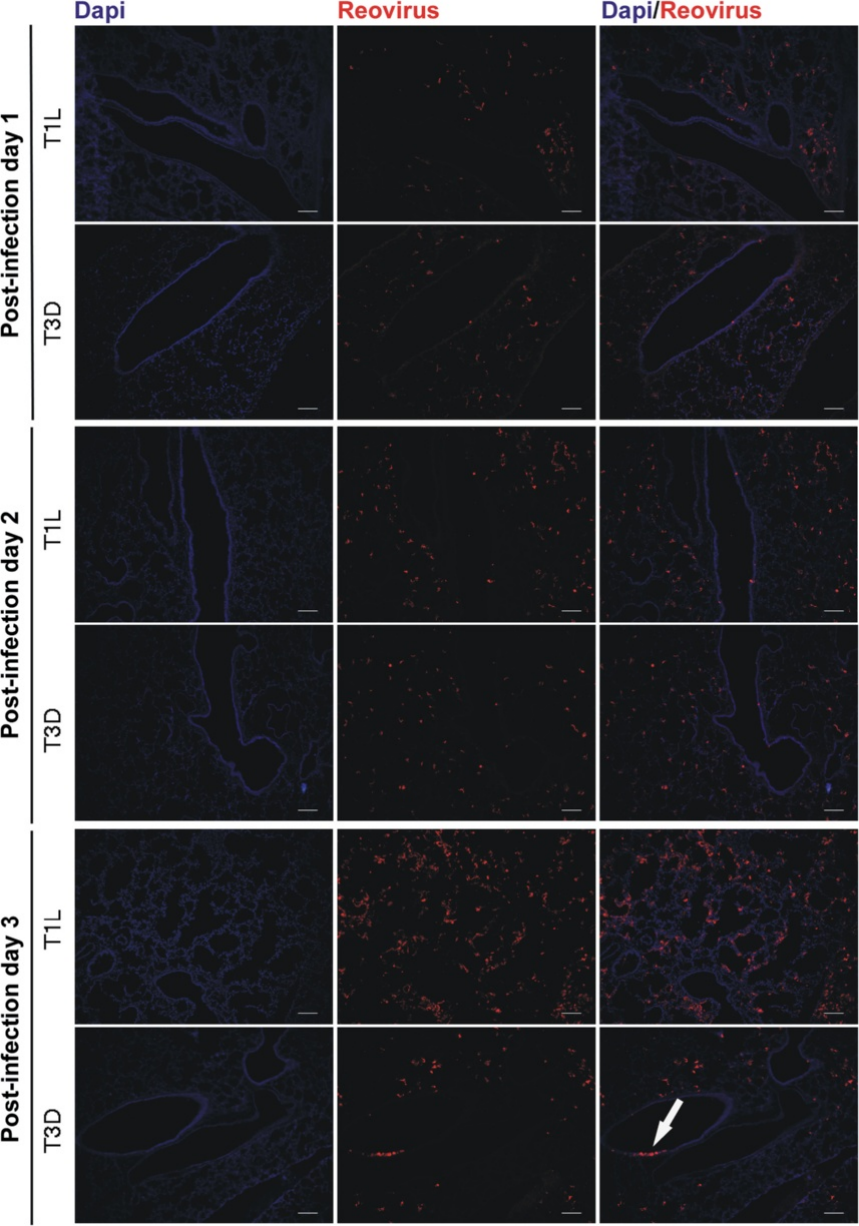
**Time course of immunofluorescent staining of virus infected suckling lung.** Two day old suckling mice were infected intranasaly with 10^5^ pfu of either T1L or T3D before fluorescent antibody staining of frozen lung sections after 1, 2, or 3 days of infection. T1L and T3D antigens were stained with virus specific antiserum as described in Figure 5. Cell nuclei were stained with DAPI, shown in blue. Size bars are 100 μM. Sagital sections show viral infection patterns similar to those of adults ( Figures 5 and 6) that again show sparse staining of epithelium in the bronchiolar airways (arrow) being restricted primarily to T3D infection.

The pattern of infection in suckling mouse lungs was very similar to adults except that more lung tissue was infected at day 3 for both virus types (Figure 7). Again the infection was largely alveolar with few infected epithelial cells, where small patches of epithelial infection were seen for T3D at day 3. Consistent with the reductions in staining after the times of peak virus yield, fluorescent antibody staining of lungs at 7 days pi showed low levels of staining of viral antigen, which was mainly associated with accumulated debris in the alveolar and bronchiolar airways in both adult and suckling mice infected with both reovirus T1L and T3D (data not shown).

### Immunofluorescent staining of brain

**Figure 8:**
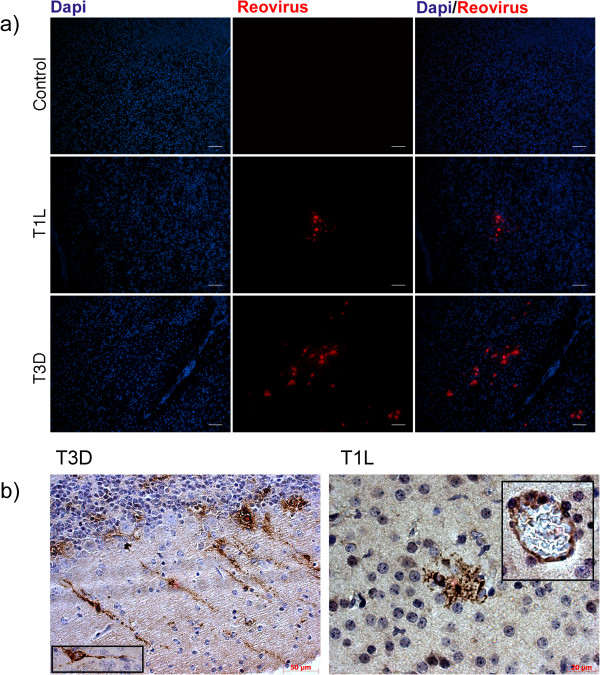
**Immunofluoresent staining of virus infected suckling mouse brain sections. a**) Two day old suckling mice were infected intranasaly with 10^5^ pfu of either T1L or T3D before fluorescent antibody staining of frozen coronal brain sections at 7 days pi. T1L and T3D antigens were stained with virus specific antiserum as described in Figure 5. Cell nuclei were stained with DAPI, shown in blue. Size bars are 100 μM. **b**) Horse Radish Peroxidase staining (in brown) of formalin fixed paraffin embedded brains of T1L and T3D infected suckling mouse brains at 7 days pi. Staining of T3D infected brains showed the presence of neurons and neuronal processes whereas the cell type infected by T1L were non-neuronal and thus lacked processes. Staining of endothelial cells in blood vessels was also observed for both T3D (not shown ) as well as T1L infected brain that is shown in insert. Images were collected with the 20X objective lens and size bars are indicated.

We also performed fluorescent antibody staining for brains of suckling and adult animals on day 7 pi. The brains of adult mice did not contain detectable levels of viral antigen which was consistent with the low levels of infectious virus at this time (data not shown). Infection of the suckling brain with T3D resulted in clusters of fluorescent cells, as shown in Figure 8a. These cell were shown to be infected neurons (including Purkinje cells of the cerebellum) with the presence of antigen in cell bodies as well as processes as shown in Figure 8b (single neuron in inset). Although brain infection with T1L was much reduced there were small areas of staining in the brainstem regions that involved non-neuronal cells with morphologies similar to activated glia (shown in Figure 8b). Staining of endothelial cells was also seen in small arterioles and capillaries of both T1L and T3D infected brains (as shown in the inset panel of the T1L infected brain, Figure 8b). Although select neuronal infection was also recorded in the hippocampus and cortex of T3D inoculated mice, further characterization is required to assess the cellular tropism and full extent of brain infection with both these viruses via the respiratory route.

Similar to the brain data, the livers of adult mice were negative by immunofluoresent staining at day 7 pi for T1L and T3D antigen (data not shown). Staining of uninfected mouse organs with anti-reovirus serum revealed no signal, as expected (data not shown). In contrast, staining of reovirus antigen at the same time point was seen in liver sections of suckling mice although this involved only a small number of hepatocytes that were more easily detectable in T1L infected than T3D infected livers (data not shown). Therefore the ability to detect antigen-positive cells in suckling brain and liver relative to adults was correlated with greater replication of mammalian reoviruses in suckling tissues than adult organs.

### Histopathology

**Figure 9:**
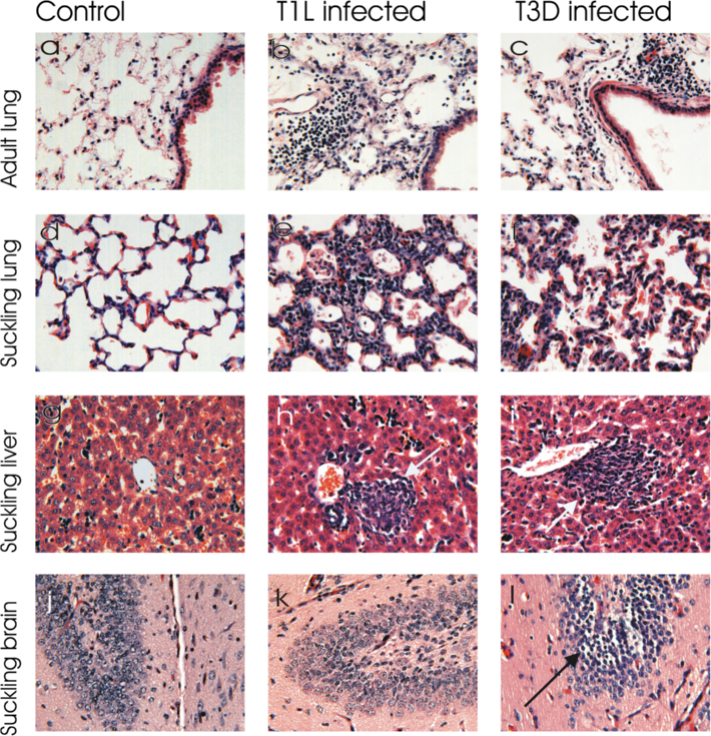
**Pathology of virus infected lung, liver and brain tissues.** Adult or 2 day old suckling mice were infected intranasaly with 10^7^ or 10^5^ pfu respectively with either T1L or T3D before sectioning formalin fixed, paraffin embedded sections for histopathological assessment by H and E. staining. Panels **a**, **d**, **g**, and **j** show uninfected control images for adult or suckling lung, liver and brain sections of mice as labeled. Panels **b**, **e**, **h**, and **k** show the T1L infected lung, liver and brain of the indicated types (suckling or adult) of infected mouse organs. Panels **c**, **f**, **i**, and **l** show the T3D infected lung, liver and brain for the indicated types (suckling or adult) of mouse organs. Arrows show local areas of cellular damage in liver and suckling brain sections that show the dentate gyrus of the hippocampus. Lungs and liver were imaged 7 days pi and brains at 13 days pi. Images were captured with the 20X objective.

We had demonstrated that respiratory inoculation with reovirus not only resulted in local infection in lung tissue but also spread to peripheral tissue sites. To assess the gross pathological consequences of infection, organs were paraffin embedded, sectioned and stained with hematoxylin and eosin at the acute stage of fatal infections; day 7 for all tissues except suckling brain that was analyzed at 13 days pi. Adult mice were infected intranasaly with 10^7^ pfu and suckling mice with 10^5^ pfu of both T1L and T3D for histopathological assessment of adult brain, lung, heart, liver, spleen, kidney, intestine and suckling, lung, brain and liver. The histological observations were consistent with the virological findings in that organs with higher viral replication (≥10^6^ pfu/g) revealed greater signs of pathology, which were detected as tissue damage and inflammation (i.e. accumulations of lymphocytes and inflammatory cells) for both T1L and T3D in lungs and livers and for T3D in sections of infected suckling brain, as shown in Figure 9. The adult and suckling lungs infected with T1L produced signs of severe patchy pneumonia associated with alveolar thickening and lymphocytic infiltration, as well as accumulation of cellular debris, with distended bronchioles and alveoli (Figure 9 panels b, c, e, and f relative to controls panels a and d). Lung infection with T3D was similar to T1L except that the degree of pathology, including areas of lymphocytic infiltration was reduced, consistent with a non-fatal primary viral pneumonia. Alveolar inflammation and thickening was more pronounced not only in suckling mice relative to adult animals but also for T1L relative to T3D infection.

Infection of the liver of adult mice was associated with some minor foci of necrosis and inflammatory cells primarily associated with the vasculature and biliary ducts (data not shown). This pattern was more extensive in suckling mice, where T1L infection was associated with detectable areas of necrotic lesions and inflammatory cells, as indicated with arrows in panels h and j of Figure 9, relative to control sections shown in Figure 9g.

The brains of adult mice infected with T1L and T3D were not significantly different from control mice at 7 days pi (data not shown). Brains of suckling mice were sectioned 13 days after infection, corresponding to the time of neurological disease symptoms in T3D infection. Brains of T1L infected suckling mice appeared normal however the brains of T3D infected suckling mice revealed areas of encephalitis associated with the hippocampus and cortical regions seen as neurons with condensed nuclei, as indicated by an arrow in panel l of Figure 9. The histopathological findings for these organs as well as the heart, intestine and spleen are summarized in Table 1. The heart and intestine appeared relatively normal, but the spleen was activated and possessed multiple follicles and white pulp. In summary, significant and severe pathology was restricted to the lungs of all mice infected with T1L and T3D viruses and the brain of T3D infected suckling mice.

**Table 1:** Histopathological assessments of adult and suckling mice following intranasal infection

**Tissue**	**T1L virus infection**	**T3D virus infection**
Adult Lung	severe patchy pneumonia, alveolar debris, lymphocytic infiltration, distended bronchioles and alveoli	mild to moderate pneumonia, discrete lymphocytic infiltration
Adult brain	appears normal	appears normal
Adult liver	few necrotic foci with cell degeneration and polymorphonuclearleukocytes	edema, necrotic foci (fewer than T1L)
Adult heart	appears normal	appears normal
Adult spleen	numerous white follicles	numerous white follicles
Adult intestine	appears normal	appears normal with some lymphocytes
Suckling lung	severe pneumonia as seen for the adult infection	moderate pneumonia
Suckling brain	minor areas of lymphocytes	encephalitis, dark staining condensed cells
Suckling liver	necrotic foci and lymphocytes	less numerous necrotic foci and lymphocytes

## Discussion

In this paper we extend the mouse models of reovirus infection to the respiratory tract where we demonstrate that both adult and suckling mice are readily infected via intranasal inoculation with prototype T1L and T3D reoviruses. Intranasal inoculation resulted in extensive lung infection that became systemic within the first day of infection. The pattern of disease was both type and age specific where T1L caused fatal, acute respiratory distress and T3D did not. In contrast, T3D produced an age-dependant fatal encephalitis in newborn but not adult mice. This indicated a uniform ability of both serotypes to initiate infection via the respiratory route but the resulting infections induced strain dependent polymorphisms with respect to extent of tissue involvement and disease type.

### The adult mouse model of respiratory reovirus infection

The overall adult mouse lung model of respiratory infection was characterized by local replication in alveolar epithelium coincident with appearance of virus in the blood stream. Within the first day of infection, blood borne virus infected peripheral organs to produce lower levels of infectious virus that were not associated with disease states. T1L was more pneumotropic than T3D and caused dose dependent fatal acute respiratory distress which occurred 4 days subsequent to the time of peak virus yield in the lung and thus appeared to be largely due to an immune mediated inflammatory response (as shown for T1L in the CBA/J mouse [20]). T3D does not replicate as well as T1L in the mouse lung (Figure 3) consistent with the findings of others in the rat lung [23].

### The suckling mouse model of respiratory reovirus infection

The suckling mouse lung model of respiratory infection was similar to the adult model for T1L but differed for T3D. Although both types replicate in the lung, T1L was more pneumovirulent than T3D causing pneumonia in both suckling and adult mice. T3D produces clinical signs of respiratory infection that were not fatal but instead resulted in dose dependent fatal encephalitis that occurred on day 12, subsequent to maximal virus replication (>10^9^ pfu/gram of brain) around day 8. The occurrence of disease subsequent to maximal virus yield again suggests a post-infectious host response to the virus as the cause of disease. Minor tissue necrosis was seen in the T1L infected liver and less so in T3D infection, which is consistent with the observation that the liver is the only site of tissue damage in the adult SCID model of intraperitoneal reovirus infection which is associated with systemic infection and hepatitis [29].

### Reovirus grows to high titre in lungs

With respect to organ tropism, both T1L and T3D strains infected the lung and all peripheral tissues examined, but T1L replicated to higher levels in the lung that was associated with its greater ability to cause acute respiratory distress in the mouse; these findings parallel those found by others in rats [23]. The clinical involvement of T1L infection with the lung was supported by the high level of infectious virus and infected lung tissue as well as the presence of interstitial alveolar inflammation. Both reovirus types grew to higher levels per gram of tissue in the suckling mouse lung that was associated with increased pneumovirulence of T1L but not T3D. Similar to adults, fatally infected T1L infected suckling mice demonstrated a time to death of 7 to 8 days, however these infections differed in dose response where fatal disease occurred with at a 100 fold reduced dose (10^5^ versus 10^7^ pfu). In contrast, suckling mice infected with similar doses of T3D lived beyond this time to subsequently die of neurologic disease.

### Lung infection primarily involved alveolar tissues

Cellular tropism in the lung was largely restricted to alveolar tissue seen as dispersed foci of infected cells similar to the infection of type I alveolar pneumocytes described for infected rat lungs [23]. Very little evidence of infection was seen for bronchiolar epithelium which was primarily detectable at later times, coincident with peak replication. The observation of alveolar rather than bronchiolar infection is opposite to the pattern seen for mouse infection with unadapted human viruses that produce local infections of the lung, i.e. influenza [28,30,31] and parainfluenza viruses [32,33]. Previous analysis of infection of mouse enteric epithelium indicate entrance of virus through the apical side of M cells [34] followed by infection of the basolateral epithelial surfaces that contain 2 known reovirus receptors, sialic acid and junction adhesion molecule [35]. Entry into the rat lung has also been shown to involve transport through M cells [24]. Proteases present in mouse lung tissue may also promote infection by generating partially uncoated virions that can enter though M cells [25] where virus could subsequently disseminate to alveolar and peripheral organ sites for productive infection, possibly via infected endothelial cells in blood capillaries as was seen in infected brain (Figure 8b). Determination of the mechanistic details of infection and spread via the respiratory route in mice requires further analysis.

### Respiratory reovirus infection resulted in systemic spread via the blood

Spread of virus in the suckling mouse has previously been shown to involve blood [4,36] as well as neurons, with T3D employing primarily the neuronal route and T1L the hematogenous route [37]. Our data are consistent with entrance of virus into the blood from the lung followed by systemic spread. The spread of virus to the liver in the first day of infection (Figure 3) must be via a fast route such as blood circulation since T3D virus can only travel 14 mm per day through nerves [37,38]. Transmission of virus via nerves may however also be operating in the respiratory suckling mouse model as T3D virus was not detected in brain until day 2 suggesting a slower mode of spread to this site of infection, possibly from the nasal cavity via the olfactory nervous system. There may also be differences in infection and/or transport of T1L versus T3D through blood vessels because T1L has been shown to have an increased ability to replicate in endothelial cells [39]. Previous analysis of intracranial injection of suckling mice showed that T1L infection was limited to ependymal cells surrounding the choroids plexus that induced fatal obstructive hydrocephalus [36,40], however we observed T1L antigen positive cells in the brain parenchyma within structures suggestive of glia (Figure 8b). This was in marked contrast to T3D that caused neuronal infection and encephalitis that was consistent with oral and intracranial infection previously reported for suckling mice [8,41]. Future studies are needed to determine the sequence of events involved in the entry and spread of reovirus from the mouse respiratory tract to peripheral tissues.

### Reovirus oncolysis

The ability of T3D to preferentially replicate in RAS transformed 3T3 cells has identified reovirus as an oncolytic virus [6,7] that has now been demonstrated to replicate in a variety of tumor types as well as treat tumors in vivo models [5,42-44]. Treatment of tumors with oncolytic viruses is a process of applied pathogenesis where viral infection is directed at tumor destruction rather than disease production. It is therefore necessary to know the pathogenesis of reovirus infection for all tissue types as a consequence of infection by various routes. A thorough understanding of reovirus infection via the respiratory route is thus of practical importance in defining the ability of reovirus to infect human tissues and cause disease or conversely for the application of reovirus to the treatment of cancer. The mouse model of respiratory infection is the only normal adult model of reovirus infection causing disease and thus extends earlier studies of pathogenesis in the infant mouse to the adult. As reovirus disease is generally more severe in the suckling mouse, the adult mouse respiratory infection may more closely represent normal human infections. Although many normal cells are resistant to infection we describe virus type and host age dependency in cellular susceptibility to reovirus infection. Indeed the increased replication of reovirus strains in suckling mice and the age dependence of encephalitis are both consistent with the preferential replication in tumors that are poorly differentiated and rapidly growing cell types. Furthermore infection with T3D did not produce disease in immunocompetent adult animals which supports its safe use as a therapeutic virus in humans.

## Abbreviations

T3D: Reovirus serotype 3 Dearing; T1L: Reovirus serotype 1 Lang; pfu: Plaque forming units; LD50: Median lethal dose; HRP: Horse radish peroxidase.

## Competing interests

All of the authors declare that they have no competing interests with respect to the publication of this manuscript.

## Authors’ contributions

Conceived and planned experiments: EGB, SB, MS. Performed experiments: LG, HL, MH, JM, EGB. Supplied reagents: EGB, SB. Wrote and edited the manuscript: EGB, MS. All authors read and approve the final manuscript.
